# Late-Onset Hypophysitis Induced by Nivolumab in Advanced Esophageal Squamous Cell Carcinoma

**DOI:** 10.7759/cureus.81806

**Published:** 2025-04-06

**Authors:** Oumaima Lamsyah, Intissar Belrhali, Yousra Al Harrak, Sihame O Lkhoyaali, Hassan Errihani

**Affiliations:** 1 Department of Medical Oncology, National Institute of Oncology, Rabat, MAR; 2 Department of Oncology, National Institute of Oncology, Rabat, MAR; 3 Faculty of Medicine and Pharmacy, Mohammed V University, National Institute of Oncology, Rabat, MAR; 4 Department of Medical Oncology, National Institute of Oncology, Faculty of Medicine, Mohammed V Faculty, Rabat, MAR

**Keywords:** esophageal cancer, hypophysitis, immune related adverse events, nivolumab, treatment

## Abstract

Immune checkpoint inhibitors (ICIs) have revolutionized cancer treatment, particularly in advanced esophageal squamous cell carcinoma (ESCC), where nivolumab has demonstrated significant survival benefits. However, these therapies may precipitate immune-related adverse events (irAEs), including endocrine disorders such as hypophysitis. While hypophysitis is more commonly associated with anti-cytotoxic T-lymphocyte-associated protein 4 (CTLA-4) agents, its occurrence following anti-programmed cell death protein 1 (PD-1) inhibitors like nivolumab remains rare and diagnostically challenging due to nonspecific symptoms and frequent absence of radiographic abnormalities.

We report a unique case of late-onset hypophysitis in a 68-year-old male with advanced ESCC, emerging after 21 cycles of nivolumab therapy. Initially asymptomatic, the patient presented with progressive fatigue, anorexia, and significant weight loss, necessitating hospitalization. Laboratory investigations identified severe hyponatremia and adrenal insufficiency, confirmed by low cortisol and adrenocorticotropic hormone (ACTH) levels. Notably, pituitary magnetic resonance imaging (MRI) revealed no structural anomalies, highlighting the diagnostic complexity of immune-mediated hypophysitis in this context.

Hydrocortisone replacement therapy led to symptomatic improvement; however, nivolumab was permanently discontinued due to unresolved adrenal insufficiency and tumor progression. This case underscores the critical need for sustained clinical vigilance and proactive endocrine monitoring in patients receiving ICIs, even in the absence of overt early symptoms. Timely recognition and intervention are paramount to mitigating life-threatening complications. Furthermore, patient education on long-term adrenal insufficiency management is essential to safeguarding quality of life.

Our report emphasizes the dual challenges of optimizing immunotherapy efficacy while managing its unpredictable risks, advocating for a multidisciplinary approach to patient care. By illustrating the unique diagnostic and therapeutic hurdles posed by delayed hypophysitis in anti-PD-1 therapy, this case contributes to the growing evidence on rare irAEs, reinforcing the importance of tailored surveillance strategies in oncology practice.

## Introduction

Esophageal squamous cell carcinoma (ESCC) remains a highly aggressive malignancy with limited therapeutic options in advanced stages. The advent of immune checkpoint inhibitors (ICIs), particularly nivolumab, has transformed the treatment landscape. In 2021, the U.S. Food and Drug Administration (FDA) approved nivolumab combined with chemotherapy for advanced ESCC based on the CheckMate 648 trial, which demonstrated significant survival benefits over chemotherapy alone [1]. Nivolumab, a programmed cell death protein 1 (PD-1) inhibitor, blocks the interaction between PD-1 and programmed death-ligand 1 (PD-L1), thereby enhancing anti-tumor immunity. However, this immune activation can trigger autoimmune toxicities, including endocrine disorders such as hypophysitis: an inflammation of the pituitary gland that often manifests insidiously with non-specific symptoms [2,3].

Hypophysitis, the second most common immune-related endocrine toxicity after thyroiditis, is frequently underrecognized due to its variable presentation. Diagnosis relies on hormonal deficiencies (e.g., adrenal insufficiency), clinical context, and imaging findings, though up to 23% of cases exhibit normal pituitary magnetic resonance imaging (MRI) [4,5]. Early detection is critical to prevent life-threatening complications like adrenal crisis.

We report a case of advanced ESCC treated with nivolumab who developed hypophysitis after 21 immunotherapy cycles (15 months), illustrating the challenges of delayed endocrine toxicity. This case underscores the imperative for vigilant monitoring, patient education, and multidisciplinary collaboration to balance oncologic efficacy with toxicity mitigation.

## Case presentation

We present the case of a 68-year-old patient who has been followed since 2022 at Émile Durkheim Hospital in Épinal, France, for bifocal esophageal squamous cell carcinoma. His medical history includes type 2 diabetes, hypertension, dyslipidemia, gout, and a history of smoking, which he quit 17 years ago. The patient presented with dysphonia for several months and dysphagia to solids. The gastroscopy performed on October 27, 2022, showed two ulcerative, proliferative tumor lesions (located at 23-26 cm and 30-36 cm from the dental arches). On November 9, 2022, a thoraco-abdomino-pelvic computed tomography (CT) scan revealed esophageal wall thickening in the thoracic region and involvement of jugular and mediastinal lymph nodes. A positron emission tomography (PET) scan on November 16, 2022, confirmed hypermetabolic tumor activity in the middle and lower thirds of the esophagus, with secondary lymphadenopathy involving the left lower paratracheal and upper mediastinal nodes, in close contact with the trachea. A bronchoscopy performed on November 14, 2022, did not reveal any endobronchial lesions. Histological examination confirmed a moderately differentiated squamous cell carcinoma with a PD-L1 expression of 3%. Due to the dual tumor localization and lymph node involvement, palliative chemotherapy with cisplatin and 5-fluorouracil was initiated following a multidisciplinary tumor board meeting on November 29, 2022. Given the PD-L1 expression (>1%), nivolumab immunotherapy (240 mg intravenously every two weeks) was added to the treatment regimen. Following six treatment cycles, a PET scan showed a complete metabolic response at the esophageal lesions, though residual moderate hypermetabolism was noted in a left laterotracheal node. Maintenance therapy with nivolumab (480 mg intravenously every four weeks) was initiated in December 2022. However, after 21 cycles, equivalent to 15 months of treatment, the patient was hospitalized in April 2024 for an overall deterioration in his state of health, marked by anorexia and important weight loss (5 kg in one month). He had episodes of nausea and diarrhea but did not report dysphagia or abdominal pain. Biological tests revealed hyponatremia (125 mmol/L), hypokalemia (3.4 mmol/L), and moderate liver cytolysis. The combination of these abnormalities raised suspicion of adrenal insufficiency, which was confirmed with a significantly low cortisol (50 nmol/L) and suppressed ACTH (<8 pg/mL) (Table 1).

**Table 1:** Laboratory measurements in our patient Hb: Hemoglobin, GFR: Glomerular filtration rate, TSH: Thyroid-stimulating hormone, ACTH: Adrenocorticotropic hormone, FSH: Follicle-stimulating hormone, LH: Luteinizing hormone, IGF-1: Insulin-like growth factor 1

Parameter	Value	Normal range
Hb (g/dl)	13	14-16.5
Sodium (mmol/l)	125	136–145
Potassium (mmol/l)	3.4	3.5–5.1
GFR (ml/min)	41	80
TSH (mIU/L)	1.26	0.3-4.0
Basal ACTH (pg/ml)	< 8	< 63
Cortisol (nmol/L)	50	138 à 690
Testosterone (ng/ml)	4.14	129–767
FSH (IU/l)	15.2	1.5–12.4
LH (IU/l)	12.3	1.7–8.6
IGF-1 (ng/ml)	86.2	55 à 212

**Figure 1:**
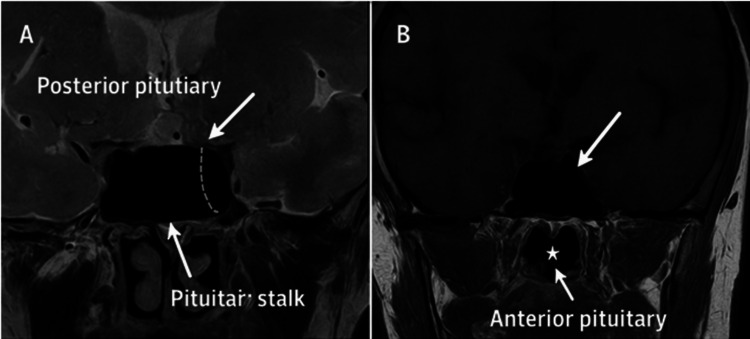
Normal pituitary MRI (A) Post-contrast T2-weighted coronal image shows posterior pituitary hyperintensity (arrow) (B) T1-weighted post-contrast coronal image demonstrates enhancing anterior pituitary (star) and stalk (arrowhead)

Through a collaborative approach with the endocrinology team, initiation of replacement corticosteroid therapy with intravenous hydrocortisone (50 mg three times a day) for 48 hours, followed by an oral tapering regimen, was achieved. Clinical improvement was rapid, with resolution of gastrointestinal symptoms and correction of electrolyte imbalances. A pituitary MRI performed on May 29, 2024, was normal without any lesions. It showed a spontaneous T1 hypersignal in the posterior pituitary, a thin and centered pituitary stalk, and a homogeneous T2 signal in the anterior pituitary with uniform enhancement after gadolinium injection (Figure 1).

Corticotropic insufficiency (cortisol: 29 µg/L, ACTH: 3.2 pg/mL) persisted, prompting continued supra-physiological dose hydrocortisone therapy (40 mg/day). This immune-related toxicity led to the permanent discontinuation of nivolumab immunotherapy, especially since recent imaging exams (PET scan and endoscopy) confirmed tumor progression. Weekly chemotherapy with paclitaxel was recommended as an alternative treatment. Additionally, therapeutic education on adrenal insufficiency was provided to the patient and his family, including the prescription of injectable hydrocortisone hemisuccinate for emergency situations. The patient was also given an adrenal insufficiency identification card.

## Discussion

This clinical case highlights the challenges associated with the use of ICIs, especially nivolumab, for the treatment of advanced esophageal cancer. Although these therapies have revolutionized the prognosis of many patients, they also expose individuals to immune-mediated adverse events, including hypophysitis, which requires heightened vigilance. The incidence of this toxicity varies according to ICI classes; combination anti-PD-(L)1/anti-CTLA-4 therapies have the highest incidence (9-10%), followed by anti-CTLA-4 monotherapy (2-6%) and anti-PD-1 monotherapy (1%) [6]. Hypophysitis typically develops within the first three to four months of anti-CTLA-4 therapy, while it appears later in anti-PD-1 treatments, with a median onset of six months [7]. Endocrine dysfunction is derived from chronic inflammatory infiltration of the pituitary, which causes progressive fibrosis. The higher incidence observed with anti-CTLA-4 therapy may be related to CTLA-4 expression in the pituitary, whereas PD-1 ligands are not detected in this gland under normal conditions. However, mutations that alter signaling pathways like phosphatase and tensin homolog (PTEN), v-raf murine sarcoma viral oncogene homolog B1 (BRAF), and epidermal growth factor receptor (EGFR) may modulate PD-1 expression and influence individual susceptibility [8-10]. Additionally, recent research has suggested the presence of PD-L1 and PD-L2 expression in murine pituitaries, reinforcing the hypothesis of selective vulnerability in ACTH-secreting cells [2]. Clinically, endocrine disorders induced by ICIs generally appear between six and twelve weeks after treatment initiation, although later-onset cases have been reported. A study on nivolumab indicated a median of 4.9 months for the development of hypophysitis and 4.3 months for adrenal insufficiency [11]. Clinical manifestations, often insidious, include fatigue, nausea, vomiting, weakness, hypotension, fever, and headaches. In contrast to hypophysitis seen with anti-CTLA-4 therapies, vision impairments are uncommon with anti-PD-1 therapies due to moderate pituitary enlargement, which is insufficient to compress the optic chiasm [12]. Hyponatremia associated with hypokalemia in the present case was a key clue to suspect corticotropic insufficiency that was confirmed by hormonal assays (Table 2).

**Table 2:** Significant correction of hyponatremia and hypokalemia following hydrocortisone replacement therapy. Values demonstrate complete normalization of both electrolytes into their reference ranges

Parameter	Pre-Treatment	Post-Treatment	Reference Range	Improvement
Sodium (mmol/L)	125	138	135-145	+13 (10.4%)
Potassium (mmol/L)	3.4	4.2	3.5-5.0	+0.8 (23.5%)

Diagnosis relies on biochemical and radiological assessment. Pituitary MRI typically reveals diffuse pituitary hypertrophy with infundibulum thickening, though 23% of patients show normal imaging despite suggestive biological abnormalities [10-14]. In our case, the MRI was strictly normal, suggesting a functional impairment secondary to the autoimmune destruction of corticotropic cells. Diagnostic criteria include the presence of at least one of the following: secondary adrenal insufficiency (low cortisol and ACTH), secondary hypothyroidism (low free T4 and inappropriate TSH), and specific pituitary abnormalities on MRI [15].

The European Society for Medical Oncology (ESMO) proposes a four-grade classification for hypophysitis severity. Table 3 (proposed addition) outlines management strategies, aligning grade-specific interventions with clinical outcomes. In mild cases (Grade 1), ICI continuation with hormonal substitution suffices. For Grade 2, temporary ICI discontinuation and oral prednisolone (0.5-1 mg/kg/day) are recommended, escalating to intravenous corticosteroids if unresponsive. Grades 3-4 require intravenous methylprednisolone (1 mg/kg) for 3-5 days, followed by gradual tapering [13]. These recommendations are supported by multivariate analyses demonstrating reduced complication rates with early corticosteroid initiation (hazard ratio 0.45, 95% CI 0.29-0.72) [13] (Table 3).

**Table 3:** ESMO grading and management of ICI-induced hypophysitis [13] ESMO: European Society for Medical Oncology, ICI: Immune checkpoint inhibitors

Grade	Severity	Clinical Features	Management
1	Mild	Fatigue, anorexia	Continue ICI + hormonal substitution
2	Moderate	Headaches, thyroid dysfunction	Pause ICI + oral prednisolone (0.5–1 mg/kg/day). Escalate to IV steroids if no improvement.
3–4	Severe/Life-threatening	Acute adrenal crisis, vision loss, coma	IV methylprednisolone (1 mg/kg) for 3–5 days → taper over 2–4 weeks. Discontinue ICI.

In cases of combined adrenal and thyroid axis involvement, corticosteroid therapy should be initiated first to prevent acute decompensation. ICI therapy can be reintroduced with careful monitoring after hormonal repletion [16]. In our patient, hydrocortisone led to rapid symptom resolution and electrolyte correction. Routine high-dose glucocorticoids remain controversial due to unproven effects on pituitary hypertrophy resolution. Endocrine function may partially recover within 4-12 weeks post-immunotherapy, but many patients require long-term replacement therapy [10].

This study has limitations inherent to single-case reports. First, our findings may not generalize to broader populations due to potential selection bias and the unique genetic/environmental profile of the patient. Second, hormonal assays (e.g., cortisol, ACTH) were performed using a single-center laboratory platform, which may introduce measurement variability compared to standardized multicenter protocols. Finally, the absence of pituitary abnormalities on MRI, while consistent with functional impairment, limits histopathological correlation.

Patient education, including "sick day rules" for corticosteroid adjustment and emergency kits, is critical for preventing acute crises [13]. This case underscores the therapeutic dilemma in oncology: balancing efficacy and toxicity. While immunotherapy initially induced a metabolic response, severe endocrine toxicity necessitated treatment reevaluation. A multidisciplinary approach involving oncologists, endocrinologists, and radiologists is essential for optimizing outcomes in patients receiving ICIs.

## Conclusions

This case highlights the critical need for rigorous monitoring in immunotherapy patients, even without overt symptoms. Unexplained hyponatremia, hypokalemia, and asthenia prompted early endocrine evaluation, revealing autoimmune hypophysitis. Management prioritized patient education (emergency hydrocortisone kit, alert card) and multidisciplinary oncology-endocrinology collaboration. While informative, this single case underscores the need for larger studies to define complication prevalence and optimize monitoring protocols. Ultimately, cutting-edge immunotherapies demand vigilance for atypical toxicities and reinforced interdisciplinary collaboration to mitigate risks.
